# Rosai-Dorfman disease manifesting as a solitary mass with fat in the thymus a case report

**DOI:** 10.1186/s13019-024-02714-x

**Published:** 2024-04-04

**Authors:** Dan Liu, Xia Liu, Yi Sha Liu, Chao Xin Zhou

**Affiliations:** 1 Department of Radiology, Sichuan Provincial People’s Hospital, University of Electronic Science and Technology of China, Chengdu, China; 2 Department of Pathology, Sichuan Provincial People’s Hospital, University of Electronic Science and Technology of China, Chengdu, China; 3 Department of Radiology, The First People’s Hospital of Liangshan Yi Autonomous Prefecture, Xichang, Sichuan China

**Keywords:** Rosai-Dorfman disease, Mediastinal mass, Fat, Computed tomography

## Abstract

**Background:**

Sinus histiocytosis with massive lymphadenopathy, also known as Rosai-Dorfman disease, is a rare, self-limiting disease that predominantly affects children and young adults. Moreover, the disease is characterized by painless bilateral cervical lymphadenopathy in 95% of the patients. However, few reports are available on the Rosai-Dorfman disease of the thymus.

**Case presentation:**

We report a rare case of thymic Rosai-Dorfman disease detected using computed tomography. During a medical examination, a 50-year-old man underwent a chest computed tomography scan, which revealed an anterior mediastinal single mass with fat in the thymus. A thymectomy was performed to completely remove the tumor using a thoracoscopic technique due to a clinical suspicion of thymoma. Furthermore, Rosai-Dorfman disease was confirmed using histological and immunohistochemical analyses.

**Conclusions:**

To the best of our knowledge, this is the sixth case of thymus-affecting solitary Rosai-Dorfman disease with histological and immunohistochemical evidence. Fat in the thymus, as was present in this case, has never been described in Rosai-Dorfman disease previously. Our results highlight the challenge of diagnosing this uncommon tumor before surgery, and more cases need to be reported to help with the preoperative diagnosis of such a rare tumor.

## Introduction

Rosai-Dorfman disease (RDD), also known as sinus histiocytosis with massive lymphadenopathy (SHML), is an uncommon illness that was initially described by Rosai and Dorfman in 1969 [1]. The disease is categorized as an idiopathic proliferative disorder of histiocytes with a presumably reactive characteristic. Rosai-Dorfman’s histologic mark, emperipolesis, describes the phagocytosis of intact immune cells by Rosai-Dorfman histiocytes. Additionally, RDD is a benign, non-neoplastic, primarily nodal-based disease [2]. Despite being typically limited to the lymph nodes, extranodal involvement is observed in 23–40% of patients with RDD, either isolated or concurrent with lymphadenopathy [3]. In the largest review of extranodal RDD by Gaitonde, the most frequent extranodal sites were identified to be skin and soft tissue (16%); nasal cavity and paranasal sinuses (16%); eye, orbit, and ocular adnexa (11%); bone (11%); salivary gland (7%); central nervous system (7%); oral cavity (4%); kidney and genitourinary tract (3%); respiratory tract (3%); liver (1%); tonsil (1%); breast (< 1%); gastrointestinal tract (< 1%) and heart (< 1%) [4]. However, only an extranodal site is rarely involved, especially in isolated thymic RDD [5].Extranodal RDD often manifests as a painless, palpable mass and compared to nodal disease has a higher incidence among women. A particular case series of extranodal diseases documented a 90% predominance of women[6]. It’s rare for it to occur in males, especially older men. Furthermore, although nodal illnesses often regress spontaneously, the course of extranodal disease is generally less indolent and can be aggressive if important organs are involved, with one case series documenting a 45% mortality rate [7].

Although a case of RDD presenting as a solitary anterior mediastinal mass with calcifications was reported by Brito et al. [8], to our knowledge, fat in thymic RDD, as in our case, has never been described previously.

Herein we reported a 50-year-old male patient with thymic RDD characterized by a solitary anterior mediastinal mass identified during a routine computed tomography (CT).The CT scan displayed multiple patchy fat depositions in the lesion and the lesion shrank mildly after half a month.

## Case presentation

**Fig. 1:**
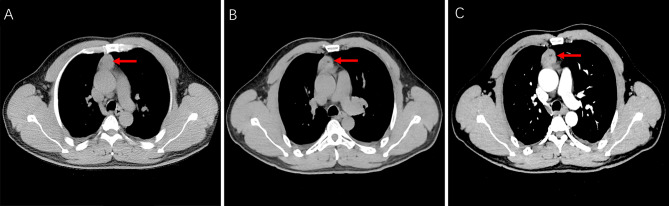
A plain chest CT scan showing a soft tissue mass in the anterior middle mediastinum, measuring approximately 2.6 cm × 2.8 cm×5.4 cm with patchy fatty component on July 28, 2023 (A); Plain and contrast-enhanced measuring 2.5 cm ×2.7 cm × 5.1 cm and showed a slight homogeneous enhancement after an intravenous contrast administration on August 14, (B, C)

A 50-year-old man was referred to our hospital for the assessment of an anterior mediastinal mass that was observed on a chest CT scan during a routine health checkup 3 months prior to presentation. He had no significant medical history or symptoms. On July 28, 2023, the patient underwent a plain chest CT scan at the First People’s Hospital of Liangshan Prefecture in Xichang City, which dysplayed a soft tissue mass in the anterior mediastinum, measuring approximately 2.6 cm×2.8 cm×5.4 cm (Fig. 1A). The mass exhibited some areas with patchy fat components with a well-defined border, and the lesion was suspected to be a thymoma. On August 14, 2023, the patient visited our facility for further evaluation. Plain and contrast-enhanced chest CT displayed an anterior mediastinal mass measuring 2.5 cm×2.7 cm×5.1 cm with regular borders, soft tissue density and patchy fat components, which also exhibited a slight homogeneous enhancement after intravenous contrast administration (Fig. 1B, C). Moreover, the patient underwent contrast-enhanced CT of the head and abdomen, which did not reveal any abnormalities. As the imagings could not confirm the diagnosis, surgery was scheduled. The patient underwent three-port video-assisted thoracic surgery (VATS) to completely remove the tumor. No invasion of the adjacent structures was observed during surgery. Following total lesion removal, the mass’s tissue was extracted using biopsy forceps for quick frozen pathology, which revealed SHML as the pathological diagnosis.

**Fig. 2:**
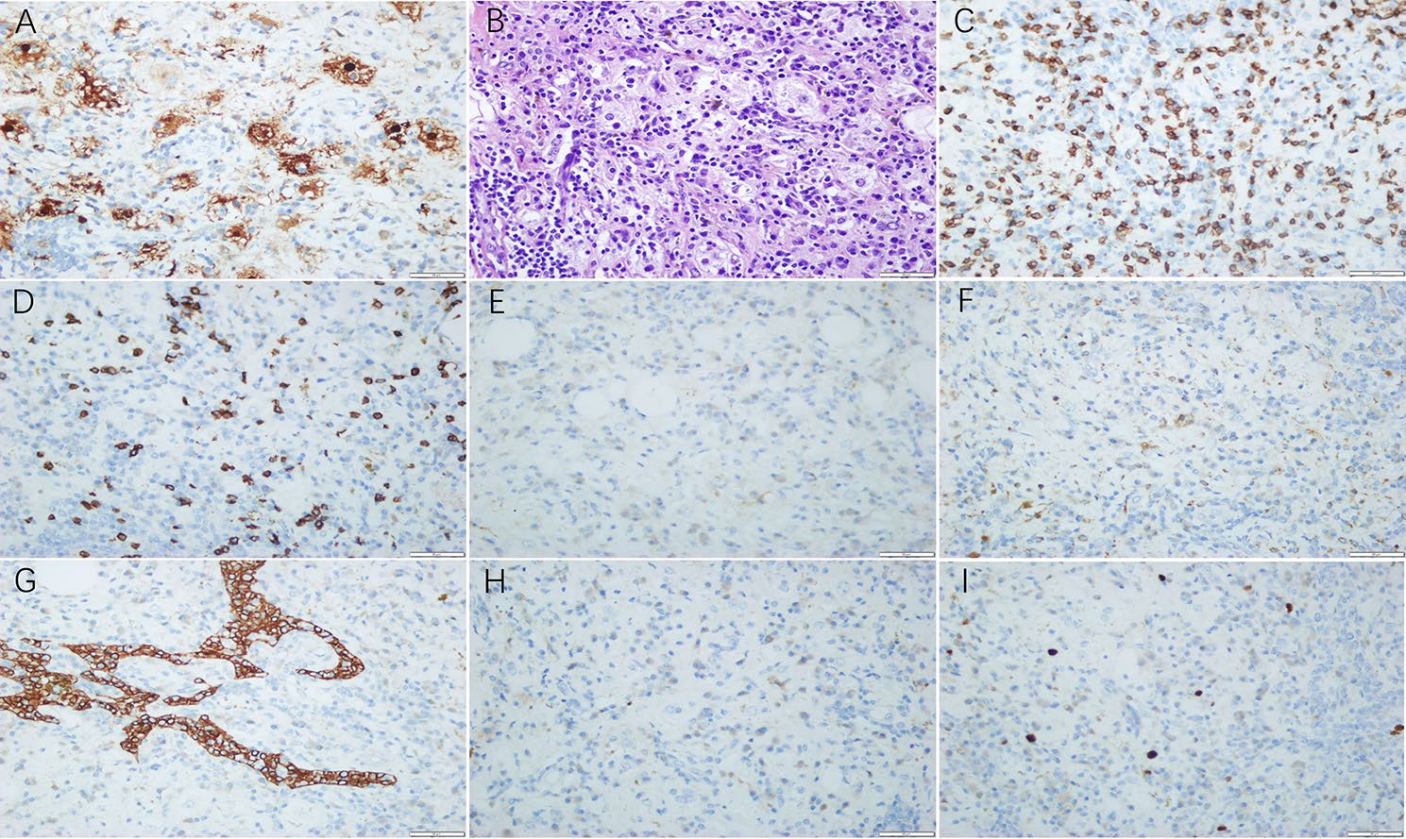
Immunohistochemistry results. S-100 protein staining was strong positive. Thymus-assosiated epithelial cell indicators CK19 was negative. CD3, CD20, CD68 were also positive, CD30, Langerin were negative; and Ki67 index was about 3% in lymphocytes and plasma cells. (a S-100 protein, b HE × 400, c CD3, d CD20, e CD30, f CD68, g CK19, h Langerin, i Ki67)

Hematoxylin and eosin (H&E) indicated the giant pleomorphic tissue cells characterized by abundant cytoplasm, vacuoles, large nuclei, and irregular nucleus were distributed in the lightly stained area. In the cytoplasm of histiocytes, emperipolesis showed as some lymphocytes and a small number of plasma cells were phagocytized occasionally with patchy fat (Fig. 2). Immunohistochemical results (Fig. 2) showed that: S-100 (+), CD68 (+), CD20 (showing B lymphocytes +), CD3 (showing T lymphocytes+), P16 (+), Pax-5 (showing B lymphocytes+), ALK(-), CD30(-), CK (-), CK19(showing B lymphocytes -), P63(-), SALL4(-), Sox-10(-), TdT(-), Langerin (-). The patient was discharged 10 days after the operation with no complication. He had been followed up for 2 months without evidence of recurrence.

## Discussion

Rosai-Dorfman disease was originally described as sinus histiocytosis with massive lymphadenopathy.This rare benign disorder is characterized by the proliferation of non-Langerhans histiocytes that exhibit emperipolesis, S100+, CD68+, CD30+, CD1a- and Langerin-. Most cases of RDD occur in children and adolescents [9]. The etiology of the disease remains unknown. Some studies have suggested that immunological dysregulation or infections with human herpes virus (HHV)-6, HHV-8, parvovirus B19, and Klebsiella are suspected causes. However, the evidence remains inconclusive [10].

The typical symptoms of RDD include painless enlarged bilateral cervical lymph nodes with fever, neutrophil elevation, elevated erythrocyte sedimentation rate, hyperglobulinemia, and other clinical symptoms [10]. However, our patient did not exhibit any symptoms, and the lesion was detected on a chest CT scan performed during a routine health checkup. This feature makes the diagnosis more challenging.

Cervical lymphadenopathy is a hallmark of RDD in 95% of the patients. Other nodal groups may also be involved, such as the inguinal (44%) and axillary (38%) lymph nodes [11]. However, RDD, is not limited to lymph nodes and extranodal organ involvement including that of the skin, bone, central nervous system, and orbital tissues and so on [4], may be observed in approximately 23–40% of cases, with a particular preference for various sites in the head and neck region [3]. Extranodal involvement may be an initial or an only presentation of this disease. RDD is rarely restricted to the thymus. Currently, few cases of RDD of the thymus have been reported, and its pathogenesis remains unclear [12, 13]. Our patient was initially misdiagnosed with thymoma because a routine chest CT revealed a solitary anterior mediastinal mass in the thymus with multiple patchy fat depositions in the lesion. This case enriches the clinical data of the disease and will help improve our understanding of the disease.

Jorge et al. reported a case of RDD that presented as a solitary anterior mediastinal mass with calcifications [8]. The patient had a suspected history of pulmonary tuberculosis.Therefore, they could speculate that calcification may have been present in the adenopathy before the development of the RDD. However, in our case, we identified numerous patchy fat depositions in the thymic RDD. To our knowledge, this case report is the first to describe fat in thymic RDD, either in the mediastinal or other nodal groups. Our patient was a 50-year-old man whose normal thymic tissue had been substantially replaced by fat. We hypothesized that the fatty tissue within the lesion was degenerative to the thymus rather than emerging from the lesion itself, suggesting gradual progression of the condition. Pathologic examination confirmed the diffuse proliferation of large histiocytic cells forming a discrete mass surrounded by thymic tissue, displaying fatty involution.

Due to the presence of fat in the anterior mediastinal mass, the differential diagnoses in our case included thymoma, lymphoma, teratoma, and Castleman disease. One should keep in mind that although RDD is a rare disorder, it is an important entity in that it may mimic other diseases.

RDD is thought to be a benign, self-limiting disease with spontaneous remission reported in up to 50% of cases [14]. The same finding was observed in our case, in which the lesion slightly reduced in size after half a month. A percentage of patients still experience prolonged disease or multiple organ invasion [15]. Currently, surgery is the primary treatment option for RDD, where total excision of the lesion is performed to promote complete remission. Refractory or nonresectable extranodal disease has historically been treated using various therapeutic approaches, such as corticosteroids, chemotherapy, low-dose interferon, antibiotics, and radiotherapy. However, the response remains highly variable, and no established standard of care for treatment is present [16].

In conclusion, the extranodal presentation of RDD poses a diagnostic challenge. Histopathological examination and imaging play important roles in the diagnosis and follow-up of RDD, which presents as nonspecific lesions. To the best of our knowledge, this case report is the first to describe the presence of fat in lymphadenopathy in RDD, either in the mediastinal or other nodal groups. Our report contributes to the clinical and imaging data on this disease, to increase awareness among clinicians, as early diagnosis of extranodal RDD is imperative for the management of the disease and improvement of prognosis.

## Data Availability

All data for this study are publicly available and are ready for the public from database of hospital.

## Abbreviations

RDD: Rosai–Dorfman disease
SHML: Sinus histiocytosis with massive lymphadenopathy
CT: Computed tomography
VATS: Video-assisted thoracic surgery
HE: Hematoxylin and eosin
HHV: Human herpes virus
